# Supplementary material for: The interactome of intact mitochondria by cross-linking mass spectrometry provides evidence for coexisting respiratory supercomplexes
Source: Mol Cell Proteomics. 2017 Dec 8;17(2):216–32. doi: 10.1074/mcp.RA117.000470 (PMC5795388; doi:10.1074/mcp.RA117.000470)
Supplement: Supplemental Data [file supp_RA117.000470_133922_0_supp_23978_fzffwf.zip › spectra_annotation/mito_spectra_annotation/57-1-10-1-9-1.pdf]

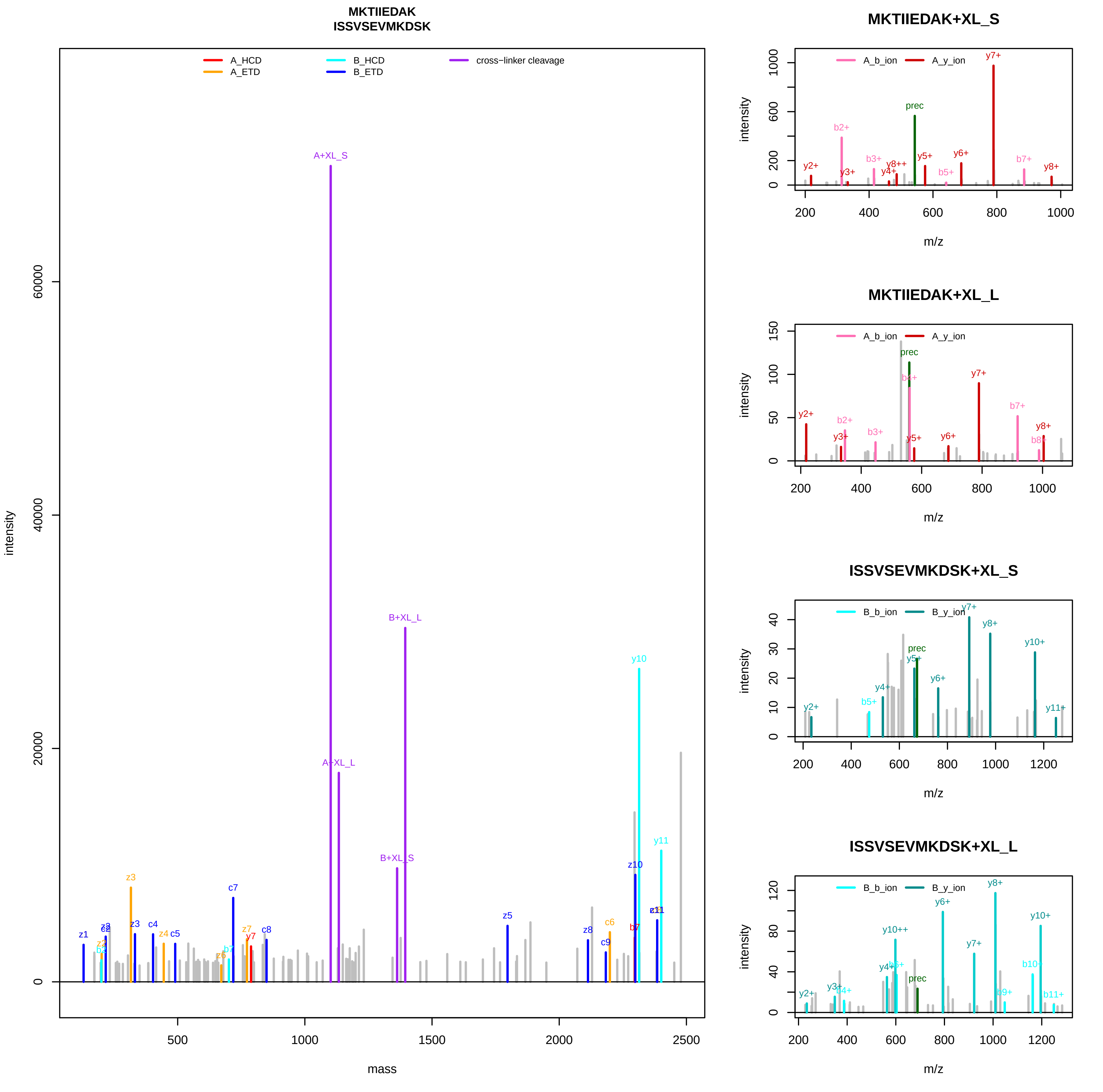
MKTIIEDAK
ISSVSEVMKDSK
A_HCD
A_ETD
B_HCD
B_ETD
cross−linker cleavage
A+XL_S
A+XL_L
B+XL_L
B+XL_S
y10
y11
z10
z1
z2
c2
z3
c4
z4
c5
c7
c8
z5
z7
y7
z8
c9
c6
b7
z11
c11
intensity
mass
0
20000
40000
60000
500
1000
1500
2000
2500
MKTIIEDAK+XL_S
A_b_ion
A_y_ion
y7+
prec
b2+
y2+
y3+
b3+
y4+
y8++
y5+
b5+
y6+
b7+
y8+
m/z
MKTIIEDAK+XL_L
b4+
b8+
ISSVSEVMKDSK+XL_S
B_b_ion
B_y_ion
y10+
y11+
ISSVSEVMKDSK+XL_L
y10++
b6+
b9+
b10+
b11+
